# Current and Potential Use of Biologically Active Compounds Derived from *Cannabis sativa* L. in the Treatment of Selected Diseases

**DOI:** 10.3390/ijms252312738

**Published:** 2024-11-27

**Authors:** Bożena Bukowska

**Affiliations:** Department of Biophysics of Environmental Pollution, Faculty of Biology and Environmental Protection, University of Lodz, Pomorska Street141/143, 90-236 Lodz, Poland; bozena.bukowska@biol.uni.lodz.pl

**Keywords:** anticancer properties, antioxidant effects, antiviral effects, anti-inflammatory properties, cannabidiol, cannabinoids, tetrahydrocannabinol

## Abstract

*Cannabis sativa* L. contains numerous compounds with antioxidant and anti-inflammatory properties, including the flavonoids and the cannabinoids, particularly Δ-9-tetrahydrocannabinol (THC) and cannabidiol (CBD). Cannabinoids have an effect on the endocannabinoid system (ECS), a cellular communication network, and are, hence, widely studied for medical applications. Epidiolex^®^, a 99% pure oral CBD extract, has been approved by the FDA for the treatment of epilepsy. Nabiximols (Sativex) is an oromucosal spray containing equal volume of THC and CBD, and it is commonly used as an add-on treatment for unresponsive spasticity in multiple sclerosis (MS) patients. Several in vitro and in vivo studies have also shown that cannabinoids can be used to treat various types of cancer, such as melanoma and brain glioblastoma; the first positive clinical trials on the anticancer effect of a THC:CBD blend with temozolomide (TMZ) in the treatment of highly invasive brain cancer are very promising. The cannabinoids exert their anticancer properties in in vitro investigations by the induction of cell death, mainly by apoptosis and cytotoxic autophagy, and the inhibition of cell proliferation. In several studies, cannabinoids have been found to induce tumor regression and inhibit angiogenic mechanisms in vitro and in vivo, as well as in two low-numbered epidemiological studies. They also exhibit antiviral effects by inhibiting *ACE2* transcription, blocking viral replication and fusion, and acting as anti-inflammatory agents; indeed, prior CBD consumption (a study of 93,565 persons in Chicago) has also been associated with a much lower incidence of SARS-CoV-2 infections. It is postulated that cannabis extracts can be used in the treatment of many other diseases such as systemic lupus erythematosus, type 1 diabetes, or various types of neurological disorders, e.g., Alzheimer’s disease. The aim of this review is to outline the current state of knowledge regarding currently used medicinal preparations derived from *C. sativa* L. in the treatment of selected cancer and viral diseases, and to present the latest research on the potential applications of its secondary metabolites.

## 1. Introduction

*Cannabis sativa* L. belongs to the family *Cannabaceae* of the order Rosales [1]. The members of this small family are typically found in the temperate regions of the northern hemisphere. *C. sativa* L. is an annual herbaceous and dioecious plant characterized by considerable genetic variation [2,3].

Cannabis is often regarded as monotypic, i.e., comprising only one species, *C. sativa* L. [4]; however, the botanical nomenclature also identifies ssp. *indica* and ssp. *sativa* as subspecies. *C. sativa* and *C. indica* differ in their THC content, with this being less than 0.3% by weight in *C. sativa* but more than 0.3% in *C. indica*; in the latter, this level is sufficient to produce a psychoactive action. In contrast, *C. indica* has a lower CBD concentration than *C. sativa* [5].

*C. sativa* L. is an easy crop to grow and, due to its high growth rate and large green leaf area, it can achieve high yields compared to other cultivated plants without the need for plant protection products and fertilizers [3]. The plants also have roots that loosen and ventilate the soil, improve its water conditions, and generally provide a beneficial environment for neighboring plants. Most importantly, they can survive with very low soil water availability and could serve as promising subjects for designing sustainable and water-saving crops [6].

*C. sativa* L. seeds are a source of oil [7,8], and various valuable essential oils can be obtained from the plants inflorescences and leaves. While *C. sativa* L. seeds are used as feed for farm animals, there is growing interest in the use of cannabis oil, meal, flour, and protein powder made from processed seeds in human nutrition [9]. *C. sativa* L. oil has great potential as a nutraceutical due to its polyunsaturated fatty acid content and because it has an ideal α-linolenic acid (omega-6) to linoleic acid (omega-3) ratio of 2.5–3:1; this value is also enhanced by the presence of bioactive CBD. The unsaturated fatty acids found in *C. sativa* L. oil, and especially γ-linoleic acid, have industrial applications in cosmetic products such as moisturizers, shampoos, massage oils, conditioners, and balms. *C. sativa* L. products are non-allergenic and have skin rejuvenating properties. The cultivation of industrial *C. sativa* L. provides a source of various products used in textiles, personal care, food, nutraceuticals, and pharmaceuticals [10,11,12].

Approximately 500 types of chemical compounds have been determined in *C. sativa* L. [3], including primary metabolites (amino acids, fatty acids, and steroids) and secondary metabolites (cannabinoids, flavonoids, stilbenoids, terpenoids, and lignans) with significant health-promoting properties [13].

When evaluating its pharmacological effects, it is essential to note that *C. sativa* L. can be further subdivided in chemotypes based on its chemical composition. The plant is currently classified into five chemotypes depending on the levels of its main phytocannabinoids: THC, CBD, and cannabigerol (CBG). While cannabis strains and chemovars often show similar phytocannabinoid levels, they are characterized by different chemical profiles, and, hence, different pharmacological effects. Cerato et al. identified several new subgroups within traditional classifications resulting from the unique composition of minor phytocannabinoids that may be essential for the evaluation of the pharmacological effects of cannabis chemovars [14]. A recent analysis of industrial *C. sativa* L. based on untargeted cannabinomics found its chemical composition to vary according to the variety and geographical location of the crop; the data also confirm that phytochemicals can play an important role in distinguishing plant samples [15].

Due to the medical potential of its cannabinoids, the medical use of *C. sativa* L. is now legal in -21 European Union countries, Canada, and 47 U.S. states [16,17]. Cannabis-based drugs are also permitted for use in treating numerous illnesses and symptoms, including cancer, AIDS, multiple sclerosis, chronic pain, nausea, anorexia, and muscle spasms, in the European Union [16]. Sales of *C. sativa* L. products in the United States have increased dramatically in recent years: in 2018, the sale of *C. sativa* L.-derived CBD products amounted to $390 million. Globally, the market for *C. sativa* L. products was estimated at $2.6 billion in 2022, and is expected to reach $3.9 billion by 2031 [18].

The aim of this article is to present the current state of knowledge regarding the current and potential use of biologically active compounds in *C. sativa* L. in the treatment of selected diseases. It takes a methodical approach to select and assess the recent literature, with most papers having been published between 2019 and 2024. Around 250 articles were initially identified in the databases, of which 157 were considered suitable for this review. The publications were identified using the PubMed, MDPI, Frontiers, Elsevier, and Springer databases, as well as Google Scholar. The review includes original research papers and reviews related to the presented topic. The search was performed with the following keywords: *Cannabis sativa;* cannabinoids; “cannabinoids and disease”; “cannabinoids and cancer”; “cannabinoids and inflammation”; “antiviral properties of cannabinoids”; “cannabinoids and SARS-CoV-2”; 9-tetrahydrocannabinol; cannabidiol; dronabinol; nabiximols; nabilone; Epidiolex^®^; Sativex and cannflavin.

This review discusses the antioxidant, anti-inflammatory, and anticancer properties of the compounds present in *C. sativa* L. It also describes currently used medicinal preparations derived from *C. sativa* L. in the treatment of selected diseases, and presents the latest research on the potential applications of its secondary metabolites.

## 2. Biologically Active Compounds in *Cannabis sativa* L.

**Figure 1:**
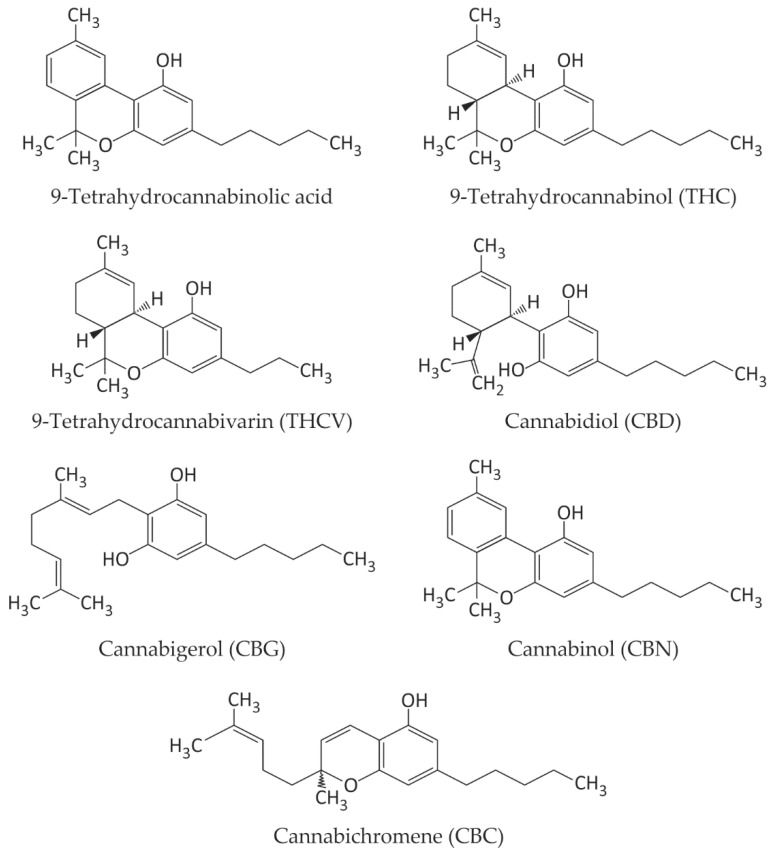
Structural formulas of cannabinoids occurring in *C. sativa* L.

*C. sativa* L. is rich in secondary compounds, including 12 fatty acids, 200 terpenes, and 20 heterocyclic compounds with nitrogen atoms in their ring structure. These are accompanied by more than 50 hydrocarbons and 125 cannabinoids [19,20]. While the most well-known cannabinoids in the species are THC and CBD, various other compounds have been isolated, including 9-tetrahydrocannabinolic acid (Figure 1) [21]. 

*C. sativa* L. oil extract is dominated by the cannabinoids CBD (59.1%) and THC (20.2%). Various monoterpenes (α-pinene, camphene, β-myrcene, and D-limonene) and sesquiterpenes (β-caryophyllene, α-bergamotene, α-humelene, humulene II epoxide, and caryophyllene oxide) have also been identified. *C. sativa* L. oil has been found to significantly inhibit tumor necrosis factor α (TNF-α) release in lipopolysaccharide (LPS)-stimulated rat monocytes [7]. Shebaby et al. [7] also demonstrated that cannabis oil extract blocked cyclooxygenase-2 (*COX-2*) and nitric oxide synthase (*iNOS*) expression induced by LPS, as well as the phosphorylation of mitogen-activated protein kinases (MAPKs). Additionally, *C. sativa* L. oil extract decreased paw edema in rats, and histopathological analysis showed reduction in inflammation and swelling.

The biologically active content of the oil was also studied in two varieties of industrial *C. sativa* L.: Kompolti and Tisza. The findings identified three terpenes, viz. isocaryophyllene, α-humulene, and β-caryophyllene β-oxide, and two terpenophenols, viz. CBD and cannabigerolic acid. Both essential oils caused cancer cell death in vitro, with that of the Tisza cultivar showing particularly marked cytotoxicity in cholangiocarcinoma cells [22].

*C. sativa* L. seeds consist of 20 to 25% protein, 20 to 30% carbohydrate, 25 to 35% oil, and 10 to 15% insoluble fiber, as well as chemical elements such as potassium, magnesium, calcium, iron, and zinc. Furthermore, they contain vitamins with highly antioxidative potential, such as A, C, E, and beta-carotene [23]. 

Oil obtained from hemp seeds is characterized by its high antioxidant potential. For example, studies on quail found those given feed with the addition of hemp seeds showed increased antioxidant activity in the blood [24]. Other laboratory studies have indicated that hempseed oil has an antiradical capacity corresponding to 1.87 α-tocopherol equivalents pel ml of oil [25]. 

*C. sativa* L. sprouts contain more biologically active compounds than their seeds. They are characterized by a higher total content of polyphenols, including flavonoids and flavanols, as well as high levels of omega-3 acids [26].

Twenty secondary metabolites have been identified in cannabis roots, including β-amyrone, glutinol, fucosterol, stigmastanol, stigmasta-3,5-diene, stigmasta-3,5,22-triene, and oleamide. The ethanol extracts of three chemovars were found to have antioxidant effects in vitro, and were identified by FRAP and ABTS. The authors propose that the antioxidant effect of the chemovars is dependent on their component compounds and is associated with external factors, such as harvest time [27].

*C. sativa* L. waxes are rich in alkanes, monoterpenes, sesquiterpenes, terpenoids, and cannabinoids, and have been attributed various healing and antioxidant effects. They are obtained from whole plants, as well as their individual fibers, seeds, and leaves, by various technological processes, with one important source being the dust [28].

### 2.1. Cannabinoids—Major Natural Ligands of CB1 and CB2 Receptors, with Anti-Inflammatory Effects

**Figure 2:**
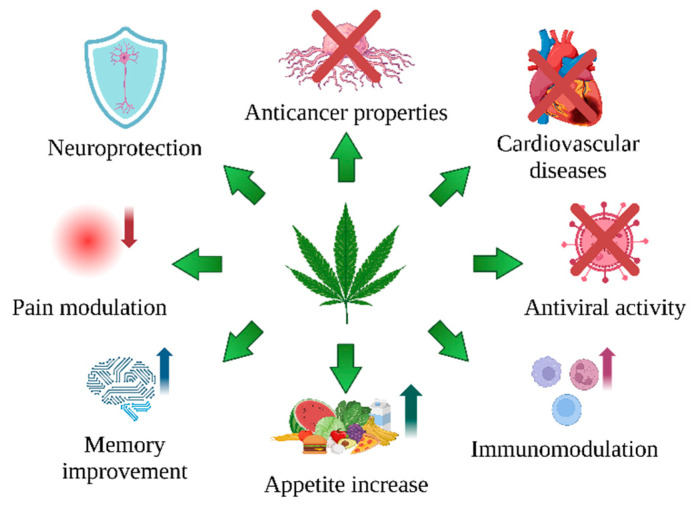
The therapeutic potential of *C. sativa* L. compounds. The up arrows indicate improved memory and increased immunity and appetite, while the down arrow indicates a pain reduction. The crossed red lines underline the potential protective role of *C. sativa* L. compounds in cancer, viruses, and cardiovascular diseases. Created in BioRender. Bukowska, B. (2024) BioRender.com/k65p423 (accessed on 1 September 2024).

Among the 125 known cannabinoids, the most represented are THC and CBD due to their high content and biological properties [20]. Recent years have shown a growth in interest in legalizing cannabis as a source of cannabinoids for treating epilepsy, multiple sclerosis, pain (migraine), or tic disorders [29,30,31]. These compounds may also be of value in treating viral diseases, e.g., SARS-CoV-2 [32]; inflammation, e.g., Crohn’s disease [33] and rheumatoid arthritis [34]; cardiovascular disorders [35]; and in cancers [36,37] (Figure 2).

The endocannabinoid system (ECS) acts as a cellular communication network that maintains many biological functions and body homeostasis. Cannabinoids themselves are known to influence numerous biological processes such as memory, pain, reproduction, bone repair, and immunity [38], and have therapeutic potential in many diseases.

The cannabinoid receptors CB1 and CB2 mediate a variety of physiological processes including the activation of MAPK phosphorylation and A-type potassium channels, and the suppression of adenylyl cyclase activity, D-type potassium, and calcium channels; these are all regarded as desirable drug targets. Both receptors are activated by endogenous endocannabinoids and the psychoactive components of marijuana [39,40].

Of the two primary cannabinoids, THC has a direct impact on cannabinoid receptors, while CBD does not. In addition, CBD does not bind to CB1 and, hence, does not have any psychoactive effect; it also does not directly trigger any receptors. However, CBD plays an important role in the endocannabinoid system. It changes the ability of receptors to bind to cannabinoids, affects other types of receptor, and increases the natural level of endocannabinoids.

THC is a partial agonist of the CB1 and CB2 receptor. In line with classical pharmacology, the response it generates appears to be strongly influenced by the expression and signaling efficiency of the cannabinoid receptors, as well as the ongoing endogenous release of cannabinoids. CBD shows an unexpectedly high potential as an antagonist of CB1/CB2 receptor agonists in cells and tissues [41].

It was reported that CBD can act as an antagonist or inverse agonist at concentrations below which it binds to either the CB1 or CB2 orthosteric sites [42]. Recently, several studies have found that CBD can act as a negative allosteric modulator of CB1: although it alters the potency and efficacy of orthosteric ligands, it does not activate the receptor itself [43,44,45]. CBD was also found to act as a partial agonist for CB2 [43]. 

CBD is considered to be of greater clinical interest than THC, partly due to its less addictive, hallucinogenic, and toxic side effects, and partly due to its anti-inflammatory, analgesic, anxiolytic, and anticancer properties, which may offer therapeutic potential in several disorders [46]. Cannabinoids exert their psychoactive and analgesic effects through CB1 receptors [41], and immunomodulatory ones through CB2 [41]. CB2 is mainly present in immune cells and plays an important role in controlling humoral and cell immune responses [47]. Targeted activation of CB2 could limit inflammation, while avoiding the psychoactive effects associated with CB1. In a clinical trial, daily cannabis treatment was associated with lower levels of pro-inflammatory biomarkers in the cerebral fluid of HIV patients [48].

THC binds with high affinity to both cannabinoid receptors as an agonist [49]. It is also an agonist of many other receptors, such as G-protein-coupled receptors and the peroxisome proliferator-activated receptor; it also serves as an antagonist of the TRPM8 transient receptor potential channel and 5-HT3 receptor A, and can increase the level of anandamide and adenosine [50]. THC suppresses the release of pro-inflammatory factors, including tumor necrosis factor α (TNF-α), interleukin-1β (IL-1β), IL-6, and IL-8, decreases nuclear factor-κB (*NF-κB*) expression, and inhibits the upregulation of cofilin-1 protein [51]. CBD and THC were found to be effective in alleviating the LPS-induced cytokine storm in human macrophages and primary HBECs, at least via modulation of NLRP3 inflammasome and STAT3 signaling pathways [52].

CBD has demonstrated anti-inflammatory effects in several conditions characterized by inflammation, including arthritis [53], neurodegenerative changes [54], and HIV [55], as well as various others [56]. Lowin et al. report that CBD increases intracellular calcium levels and reduces cell viability and IL-6/IL-8/MMP-3 production of rheumatoid arthritis synovial fibroblasts [53]. Recent studies have suggested that CBD is able to reduce the formation of lipid inflammation precursors (e.g., arachidonic acid) and cytokines in rat cerebral cortices, this being a primary event in the development of neurodegenerative diseases [54]. CBD has also been found to reduce the production of various inflammatory cytokines and chemokines in HC69.5 human microglial cells following HIV infection compared with THC. Treatment resulted in the reduction in inter alia MIF and SERPIN E1, IL-6, IL-8, and IL-1 β, as well as GM-CSF, MCP-1, CXCL1, and CXCL10 [55].

Cannabinoids inhibit pro-inflammatory cytokine activity by the effect of the CB1 and CB2 receptors. As such, CBD offers potential for reducing the inflammation associated with Crohn’s disease and ulcerative colitis [33]. Cannabinoids can also be used to alleviate rheumatoid arthritis by regulating the immune response [34]. CBD and THC ameliorate autoimmune effect on the nervous system, and are, hence, used to treat severe spasticity and pain in MS [57]. They have also demonstrated potential in the treatment of systemic lupus erythematosus [58], type 1 diabetes [59], and various types of neurological disorders, e.g., Alzheimer’s disease [60].

### 2.2. Antioxidant Compounds

Antioxidant substances, like phenolic compounds, tocopherols, and phytosterols, have been identified in *C. sativa* L. Those compounds have been long recognized to exhibit antioxidant properties in plants [6]. One study based on targeted metabolomics found that the metabolomes of seven cannabis varieties consisted of a total of 1001 metabolites, including 201 flavonoids, 86 alkaloids, and 149 phenolic acids [61].

Among polyphenols, a variety of flavonoids have been detected, such as apigenin, luteolin, kaempferol, quercetin, rutin, and methylated flavones like cannflavin A (CFL-A), B (CFL-B), and C (CFL-C), as well as the structural isomer of CFL-B, isocanflavin B (IsoB) [62,63]. Polyphenols are known to be powerful antioxidants that neutralize free radicals by donating an electron or hydrogen atom, and by chelating transition metals [64].

Barrett et al. found cannflavins A and B to inhibit the release of prostaglandin E2 (PGE2) from human rheumatoid synovial cells 30 times more effectively than aspirin under ex vivo conditions. Both cannflavins A and B demonstrate potent anti-inflammatory activity via the inhibition of PGE2 release induced by 12-o-tetradecanoylphorbol 13-acetate and microsomal prostaglandin E synthase-1 [65,66]. A review by Erridge found cannflavins (CFL-A) to have antioxidant properties in preclinical study models (carried out on laboratory animals or cell cultures), and to have antiparasitic (CFL-A and CFL-C), neuroprotective (CFL-A), and anticancer (isocannflavin B and CFL-B isomer) properties. In silico screening found CFL-A to have binding affinity for viral proteins [67] and to inhibit transforming growth factor beta-activated kinase 1, an important therapeutic target in inflammation-related diseases [68]. In vivo experiments showed a significant increase in the survival of male and female mice with pancreatic cancer compared to control cohorts after the use of the cannflavin B isomer (FBL-03G) [69].

Hemp seeds are rich in several phenolic compounds, including phenolic acids, lignamides, phenolic amides, and flavonoids [70]. The most abundant phenolics present in hemp flour samples were found to be hydroxycinnamic and protocatechuic acids [71]. 

Furthermore, *C. sativa* L. seeds have been found to contain a number of phenylpropionamides and their random oxidative coupling derivatives, i.e., lignanamides which significantly inhibited proliferation in a U-87 glioblastoma cell line by inducing apoptosis and suppressing autophagic cell death [72]; they also protected against nerve inflammation in a rat model of streptozotocin-induced Alzheimer’s disease, which was attributed to the regulation of primary bile acid biosynthesis and taurine and hypotaurine metabolism [73]. In a mouse model, an extract containing fourteen phenylpropionamides prevented the learning and spatial memory damage induced by lipopolysaccharide [74].

Other bioactive substances include tocopherols that exhibit antioxidant effects by scavenging lipid peroxide radicals, which promote lipid peroxidation and protect unsaturated fatty acids from oxidative damage [75]. 

Also, the peptides derived from cannabis proteins have been found to be strong antioxidants, which can be employed in both the phytomedicine and food industry [76]. Wang et al. found bioactive *C. sativa* L. protein hydrolysates to have both anti-inflammatory and antioxidant properties in LPS-stimulated BV2 microglial cells, inhibiting the progression of inflammation associated with the NF-κB and Keap1-Nrf2 signaling pathways [77]. *C. sativa* L. proteins also demonstrate various immunomodulatory effects. Rodriguez-Martin et al. found treatment with hemp protein products to decrease certain pro-inflammatory mediators (TNFα, IL-1β, and IL-6) and increase anti-inflammatory mediators (IL-10 and IL-4) in lipopolysaccharide (LPS)-activated primary human monocytes [78].

Extracts from the flowers [79,80], leaves [81,82], roots [82], and seeds [83] of *C. sativa* L., and their oil [84,85], contain a rich composition of antioxidants, or single compounds (e.g., CBD or THC) [86]; these have been found to counteract the production of free radicals (Table 1). Recent studies have also shown that the digestion of hulled *C. sativa* L. seeds increases the inhibition of radical scavenging by these compounds [83]. THC and CBD have similar antioxidant activity to vitamins E and C [87,88]. The phenolic groups readily oxidized to quinoid forms, and the unsaturated bonds found in non-olivetolic fragments of these cannabinoids could be responsible for their antioxidant properties [39,87].

Cannabinoids also act as indirect antioxidants, capable of modulating the redox balance by increasing the level of reduced glutathione (GSH) [89,90]; activating antioxidant enzymes such as superoxide dismutase (SOD) [91], catalase (CAT), glutathione peroxidase (GPx), and glutathione reductase (GR) [85,91]; and by suppressing pro-oxidant enzymes [56] (Table 1).

**Table 1:** Antioxidant activity of extracts, oil, and single compounds contained in *C. sativa* L.

Extract/Compounds	Tested System	Concentrations	References
*C. sativa* L. extracts and cannabinoids decrease free radicals’ level			
*C. sativa* L. essential oil (β-caryophyllene, α-humulene, β-myrcene, α-pinene)	Chemical system	IC_50_ = 0.98 mg/mL for DPPH EC_50_ = 1.74 mg/mL for FRAP 0.101 mg AAE/g EO for TAC	[84]
*C. sativa* L. extracts of flowers (flavonoids, terpenoids, saponins, anthocyanins, tannins, and reducing sugars)	Chemical system	for DPPH Ethanol extract—IC_50_ = 0.23 mg/mL Hexanic extract—IC_50_ = 0.38 mg/mL Chloroformic extract—IC50 = 0.77 mg/mL	[79]
*C. sativa* L. extract (β-caryophyllene, its oxide, CBD, THC, α-pinene, α-humulene, 2-monolinolein, methyl eicosatetraenoate and γ-sitosterol)	Chemical system	for DPPH (at different vegetative stages) Leaf—15.03 to 35.04 mmol/L extract Roots—0.29 to 1.56 mmol/L extract	[82]
Hulled *C. sativa* L. seeds and their compounds after digestion (in vitro)	Chemical system	Percentage inhibition of radical scavenging activity (Pi) 39.97 ± 0.71% after the oral phase 50.28 ± 0.62% of the intestinal phase	[83]
*C. sativa* L. extract	In vitro— neuro-2a cell line	For DPPH IC_50_—0.06 mg/mL for lyophilized aqueous extract IC_50_—0.10 mg/mL for the residual biomass hexane extract) IC_50_—0.005 mg ml/L for ascorbic acid	[80]
*C. sativa* L. oil 1 mg oil/kg BW for 3 weeks	In vivo— rats with a sucrose-rich diet	For ROS levels in the liver Rats (SRD)—2.46 arbitrary units Rats (SRD + Ca)—1.45 arbitrary units	[85]
Cannabinoids prevented the oxidation of DHR and H_2_DCF.			
THC and CBD	In vitro— aged pancreatic islet cells	0.1–100 µmol/L	[86]
Cannabinoids: CBG, CBD, Δ9-THC, CBN, CBGA, CBDA and Δ9-THCA	chemical system	EC_50_ CBD—11 mmol/L EC_50_ THC—18 mmol/L EC_50_ BHT—18 mmol/L	[92]
THC and CBD	In vitro— rat neuronal cell cultures	EC_50_ of 2–4 µmol/L	[39]
CBD	In vitro—rat PC12 cells with β-amyloid induced toxicity	0.1–100 µmol/L	[93]
*C. sativa* L. oil and cannabinoids reduce the lipid peroxidation			
CBD	In vitro— rat PC12 cells with β-amyloid-induced toxicity	Decrease in MDA level in PC12 caused by β-amyloid 0.1–100 µmol/L	[93]
*C. sativa* L. oil 1 mg oil/kg BW for 3 weeks	In vivo— rats with a sucrose-rich diet	For TBARs in serum Rats (SRD)—3.94 μmol/L Rats (SRD + Ca)—2.99 μmol/L	[85]
CBD intraperitoneal injection	In vivo— Rats with traumatic spinal cord injury	2.5–20 mg/kg	[89]
CBD	In vivo— rats	50, 100, 200 ng/rat	[91]
*C. sativa* L. oil and cannabinoids increase the level of GSH			
CBD	In vivo— rats chronically irradiated with UV	120 mg/kg BW	[90]
CBD intraperitoneal injection	In vivo— rats with traumatic spinal cord injury	2.5–20 mg/kg	[89]
Cannabis oil 1 mg oil/kg BW for 3 weeks	In vivo— rats with sucrose-rich diet	For GSH levels in the liver Rats (SRD)—45 ηmol/mg protein Rats (SRD + Ca)—61 ηmol/mg protein	[85]
*C. sativa* L. oil and cannabinoids elevated the activity of antioxidative enzymes: SOD, CAT or/and GPX and GR			
CBD	In vivo— rats	Injected at 50, 100, and 200 ng/rat for five consecutive days	[91]
Cannabis oil 1 mg oil/kg BW for 3 weeks	In vivo— rats	For GPx and GR activities in the liver Rats (SRD)—85 and 28 mU/mg Rats (SRD + Ca)—147 and 44 mU/mg	[85]

AAE: ascorbic acid equivalents; CAT—catalase; DPPH—2,2-difenylo-1-pikrylohydrazyl; BW—body weight; FRAP—ferric reducing antioxidant power; DHR—dihydrorhodamine; EO—essential oil; GSH—reduced glutathione; GPX—glutathione peroxidase; GR—glutathione reductase H_2_DCF—2′,7′ dichlorofluorescein; SOD—superoxide dismutase; SRD—rats with sucrose-rich diet; SRD + Ca—rats with SRD and oil; TAC—total antioxidant capacity.

### 2.3. Potential Use of Biologically Active Compounds Derived from Cannabis sativa L. in the Treatment of Cancers

Oxidative stress, and the consequent imbalance in the redox system and inflammation, plays an important role in many conditions, including cancer, inflammatory diseases, and neurodegenerative diseases. The various antioxidant components of *C. sativa* L. interrupt the chain reactions of free radicals by capturing them or converting them to less active forms; they also ameliorate oxidative conditions, preventing the formation of radicals [94].

As some conditions, including cancer, can alter the endocannabinoid system, the use of THC and CBD as anticancer agents has become a promising research area. Although it is widely accepted that conventional therapies, such as chemotherapy and radiotherapy, have more side effects in adult cancer patients then cannabinoid treatment, these results have not been translated into many clinical trials for adults. Nevertheless, interest in these compounds in Medicine and Oncology has been growing [95]. Cannabinoids are mostly used as support for patients receiving radiation or chemotherapy: they alleviate many of the common symptoms and side effects connected with the treatment of cancer [96]. 

Currently, little evidence exists regarding the effective treatment of cancer in animals, with only several epidemiological studies examining the effects of cannabinoid administration; as such, the preclinical model of cancer demands more solid evidence to confirm whether cannabinoids have anticancer effects in humans [36,97,98]. An in silico study docked six cannabinoids from *C. sativa* L. with MAPK-ERK signaling pathways to identify their possible binding interactions; among these, CBD and THC exerted the greatest binding affinities with proteins of the MAPK-ERK signaling pathways, and their pharmacokinetics and drug-likeness-related profiles suggest that these cannabinoids could be potential inhibitors in cancer treatment [99].

**Figure 3:**
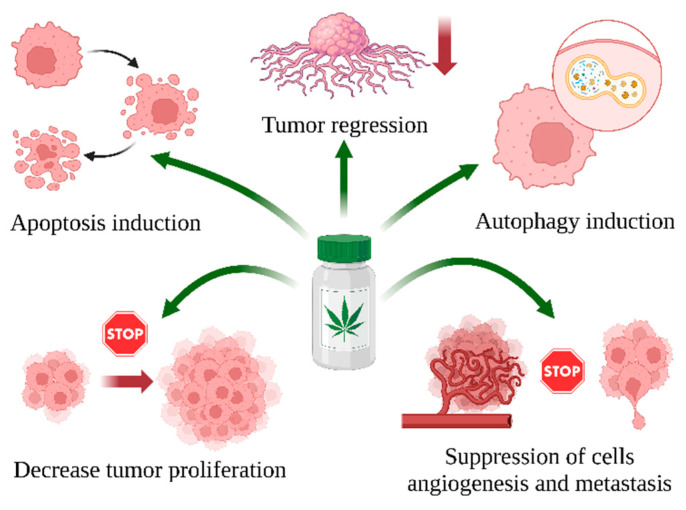
Mechanism of anticancer properties of *C. sativa* L. compounds by induction of apoptosis (e.g., by increase caspase 3/7 activity, upregulated *TP53* and *Bax*—expression [100]), induction of autophagy (e.g., upregulates LC3BII, downregulates p62,and inhibits p-PI3K, p-AKT, and p-mTOR pathways [101]), induction of tumor regression (e.g., decreases colony-stimulating factor 1 (CSF-1) level [102], induces DNA fragmentation, and arrests cell progression at the G1 cell cycle checkpoint) [103], decrease in tumor proliferation (e.g., by inhibiting ERK1/2 signaling pathway phosphorylation [104]), and suppression of cell invasion (e.g., by increased expression of *TIMP-1*, which mediates an anti-invasive effect [105]) in cells and animals. Created in BioRender. Bukowska, B. (2024) BioRender.com/p40b597 (accessed on 1 September 2024).

Studies have revealed that cannabinoids may have a different mechanism of anticancer potential in various cancer cell types in vitro and in animals in vivo (Figure 3).

The in vitro research has included breast cancer cells [106], melanoma cells [96], lung cancer cells [97], and prostate cancer cells [107], while the in vivo studies include brain glioblastoma in animals [108,109], malignant melanoma tumors in mice [96], and metastatic melanoma in NSG mice [110]. Clinical trials have also been conducted [36,37]. 

Most importantly, THC and CBD, and other phytocannabinoids, have been revealed to trigger apoptosis, inhibit cancer cell proliferation, and modulate angiogenesis and metastasis [38,111,112] (Table 2).

#### 2.3.1. Lung Cancer Cells/Lung Cancer

Ramer et al. [97] evaluated the role of COX-2 and PPAR-γ in the pro-apoptotic and regressive effects of cannabinoids in human lung cancer cells (A549, H460). They noticed that cannabinoids decreased the viability of cancer cells by triggering apoptosis, *COX-2* and *PPAR-γ* genes upregulation and their proteins expression. Cancer cell lines treated with cannabidiol demonstrated elevated levels of COX-2-dependent prostaglandins; in particular, PGD(2) and 15-deoxy-Δ(12,14)-PGJ(2) translocated PPAR-γ to the nucleus, and triggered PPAR-γ-dependent apoptotic cell death. Additionally, in A549-xenografted nude mice, cannabidiol induced upregulation of *COX-2* and *PPAR-γ* genes and their proteins in tumor tissue and tumor regression. Studies on A549 cells from human lung cancer have found CBD to decrease tumor invasion and metastasis, and inhibit cell growth [97,117,118].

CBD also slowed tumor invasion by human lung carcinoma cells (A549), human cervical carcinoma (C33A), and HeLa [105]. The authors of this study assessed the effect of THC and the stable anandamide analog R(+)-methanandamide (MA) on h expression of matrix metalloproteinases and endogenous tissue MMP inhibitors (TIMPs) in A549 cells. The role of TIMP-1 was determined by transfecting HeLa, C33A, or A549 cells with siRNA targeting TIMP-1. It was found that MA and THC induced a time- and concentration-dependent suppression of HeLa cell invasion through Matrigel, and that this was assisted by elevated *TIMP-1* expression; such elevated *TIMP-1* expression appeared to mediate the anti-invasive effects of cannabinoids. Ramer and Hinz, thus, recommend cannabinoids in the treatment of highly invasive cancers [105].

An in vivo study of a Lewis lung cancer grafted mouse model by Xu et al. [116] found that *C. sativa* L. essential oil (HEO) significantly inhibited tumor growth; it also downregulated the levels of tumor inflammatory cytokines TNF-α and IL-6, modified the immune organ index, and increased the numbers of immune-related CD4+ and CD8+ T lymphocytes and the CD4+/CD8+ ratio.

#### 2.3.2. Hepatocellular Carcinoma Cells

Recently, Lee et al. studied the effects of a combination of cabozantinib and CBD therapy on hepatocellular carcinoma (HCC) cells. Their data indicate that cannabidiol could increase the death of apoptotic cells by cabozantinib (a multi kinase inhibitor) through phosphorylation of p53, which is regulated by ER stress in HCC cells. The addition of CBD increased the anticancer activity of cabozantinib, which the authors attribute to increased apoptosis and cytotoxicity [113].

#### 2.3.3. Prostate Cancer Cells/Prostate Cancer

In other research, cannabis extract, cannabidiol, and cisplatin was found to cause antiproliferation of PC3 cancer cells by increasing caspase 3/7 activities. The study also showed apoptotic changes in studied cells when retinoblastoma binding protein 6 (RBBP6) was silenced, with upregulation of *TP53* and *Bax* expression and downregulation of *Bcl2* expression. In other experiments, it was observed that the tumors in the mice had decreased in size after treatment with cisplatin and CBD. CBD, hence, appears to be a viable therapy for treating prostate cancer cells when combined with the silencing of RBBP6 [100].

CBD seems to induce cell death mostly through the intrinsic apoptotic pathway. This is connected with the dysregulation of the Bcl-2 family of proteins, resulting in mitochondrial dysfunction (i.e., depolarization and ROS production). Cannabinoids can target various mitochondrial processes by modulating multiple complex signaling pathways; the targets include intracellular calcium regulation, the bioenergetic metabolism, and mitochondrial apoptosis and dynamics, such as mitochondrial fission and fusion, transport, mitophagy, and biogenesis [119].

An open-label, single-center Phase I/Ib trial was performed to determine the safety and initial efficacy of CBD using Epidiolex^®^ in 18 patients with biochemically recurrent prostate cancer. Epidiolex^®^ 800 mg/day was observed to be well tolerated, and to have an acceptable safety profile in these patients [107]. Unfortunately, there are inherent limitations to this study, such as the short duration of treatment with Epidiolex, the small sample size, and the lack of a comparator group.

#### 2.3.4. Melanoma Cancer Cells/Malignant Melanoma Tumors

A study on a mouse model found cannabiberrol (CBG) to inhibit tumor progression and reduce the amount of macrophages associated with the tumor [102]. Combining CBG with the anti-programmed cell death ligand 1 (αPD-L1) further reduced tumor progression, increased survival, and increased the infiltration of active toxic T cells. Furthermore, the cannabis extract treatment also depleted colony-stimulating factor 1 (CSF-1) secretion by melanoma cells. The authors propose that CBG employs a novel mechanism to modulate the tumor microenvironment and increase immune checkpoint blockade therapy; as such, it offers promising therapeutic potential in various tumors treated with elevated expression of colony-stimulating factor 1.

Another study found *C*. *sativa* L. (PHEC-66) extract to trigger apoptosis in MM418-C1, MM329, and MM96L melanoma cell lines [103]. This was associated with increased expression of anti-apoptotic markers (i.e., *Bax*) and a decrease in anti-apoptotic markers (i.e., *Bcl*-2-). Additionally, PHEC-66 caused DNA fragmentation and stopped cell progression at the G1 cell cycle control point. These results suggest that PHEC-66 may have potential as an adjunct therapy for the treatment of malignant melanoma.

Simmerman et al. [96] assessed the effects of cannabinoid derivatives on malignant B16F10 murine melanoma tumors. The tumors were subcutaneously implanted in C57BL/6 mice, which were then treated with 5 mg cisplatin and 5 mg CBD. The mice treated with CBD demonstrated significantly smaller tumor sizes compared to the controls. Mice treated with cisplatin exhibited the longest survival period, while those treated with CBD demonstrated improved quality of life and movement. While cisplatin induced the strongest tumor growth suppression in all experimental groups, CBD significantly delayed tumor growth compared to controls, and subjective observations suggested that CBD was better tolerated.

Recent research has examined the pro-apoptotic effects of cannabinoids in metastatic melanoma in vivo and in vitro. Various melanoma cell lines were treated with cannabinoids at various concentrations, and their efficacy in combination with trametinib was assessed in vivo in NSG mice. Cannabinoids were noticed to deplete cell viability in the multiple melanoma cell lines in a concentration-dependent manner, triggering apoptosis by releasing mitochondrial cytochrome c and activating various caspases. These substances substantially depleted tumor growth in NSG mice and were as potent as trametinib [110].

Another study evaluated the expression of genes in A375 human melanoma cells after treatment with *C. sativa* L. extracts. It was observed that a mixture of THC and CBD of *C. sativa* L. fractions triggered apoptosis in the cancer cells. During apoptosis, a number of genes were upregulated, including DNA damage-induced transcript 3, colony-stimulating factor 2, and E2F transcription factor 1. The mixture inhibited ERK1/2 signaling pathway phosphorylation, which is responsible regulating the proliferation of A375 melanoma cells. In addition, the mixture of THC and CBD disrupted melanoma cell migration [104].

#### 2.3.5. Glioblastoma and Neuroblastoma Cells/Malignant Gliomas

Growing evidence indicates that cannabinoids can be successfully employed in glioblastoma (GBM) treatment [109]. A few of in vivo studies have shown that cannabinoids are able to substantially deplete tumor volume in orthotopic and subcutaneous animal models of glioblastoma [120,121]. Others indicate that the cytotoxic effects of CBD in human and canine glioblastoma cells resulted from dysregulation of calcium homeostasis and mitochondrial activity [122]. Synthetic cannabinoids (WIN55,212-2 or JWH133) induce autophagy by the accumulation of LC3-II and mitochondrial apoptotic pathways in human glioblastoma cells (T98G and LN229), irrespective of any deficiency in *TP53* or *PTEN* tumor suppressors [123].

CBD was found to trigger autophagy in a human neuroblastoma cell line (SH-SY5Y) by regulating the phosphorylation of ERK1/2 and AKT kinases; this process involved ULK1, but in an mTORC1-independent pathway. The activation of these signaling pathways involves the cannabinoid receptors CB1 and CB2, as well as TRPV1 receptors, which are essential for promoting neuronal cell survival and proliferation [124].

Zhong et al. [114] assessed the anticancer effect of cannabinol on A172 glioblastoma, HepG2 liver cancer, and HCC1806 breast cancer cell lines. Cannabinol (CBN) induced apoptosis by p21 and p27 downregulation and an arrested cell cycle in the G1 or S phase by reducing CDK1, CDK2, and cyclin E1 levels. It was also revealed that CBN decreased the proliferation of the studied cancer cells and modulated the levels of cannabinoid receptors including GPR18, CB2, and GPR55.

Cannabinoids are characterized by the ability to inhibit tumor angiogenesis. Blázquez et al. [36] observed that local administration of the nonpsychotic cannabinoid JWH-133 in mice inhibited the angiogenesis of malignant gliomas. It was revealed that while the treated tumors were characterized by small and impermeable blood vessels, those of the untreated tumors were large and porous. Hence, tumor size may be influenced by the biological activities of the cannabinoids.

#### 2.3.6. Studies on Humans—Clinical Trials

Although *C. sativa* L. compounds have been demonstrated to have clinical efficacy in preclinical studies, clinical trials with oncology patients are lacking.

An analysis of 207 preclinical articles, including 77 unique case reports, found no strong clinical trial data to confirm that *C. sativa* L. compounds may have benefits against various cancers: breast, central nervous system, gynecological, leukemia, lung, prostate, and pancreatic cancer [125]. Further prospective and randomized studies on this clinically important topic are needed to draw clear conclusions. 

Two clinical trials have examined the effect of cannabinoids on gliomas. The first, a pilot study, covered nine patients [36], and the second, a phase II study, was realized by GW Pharmaceuticals on 21 adult patients (clinical study NCT01812603) [37]. Patients taking THC:CBD plus temozolomide (TMZ) demonstrated an 83% one-year survival rate, with a median survival of over 662 days (369 days in the control group). The patients in the control group received only TMZ and achieved a 44% one-year survival rate.

### 2.4. Antiviral Effects of Cannabinoids

**Figure 4:**
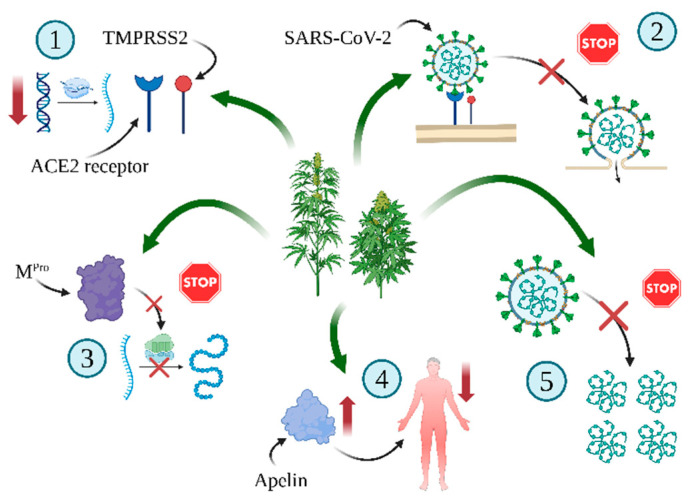
Molecular mechanism of antiviral effects of *C. sativa* L. compounds. (1) Downregulating serine protease TMPRSS2 and lowering *ACE2* transcription levels [130], (2) preventing SARS-CoV-2 spike protein-mediated membrane fusion [131], (3) blocking the virus translation and replication by suppressing enzyme of SARS-CoV-2 i.e., M^pro^ protease (in silico and in vitro experiments) [132], (4) upregulation of apelin level [133], and (5) blocking viral replication, in part by upregulating the host IRE1α RNase endoplasmic reticulum (ER) stress response and interferon signaling pathways [32]. Created in BioRender. Bukowska, B. (2024) BioRender.com/k57l772 (accessed on 1 September 2024).

The antiviral properties of cannabinoids [126,127,128] have been tested in diseases such as SARS-CoV-2 [32] and HIV [129]. The tested cannabinoids were found to inhibit severe viral infection of the SARS-CoV-2 virus by different mechanisms (Figure 4). 

In SARS-CoV-2 infection, the virus enters the host via ACE2, which is present in several tissues, including the oral and nasal membranes, lung tissue, renal tissue, and gastrointestinal tract. The expression of *ACE2* in the nose epithelium is age dependent, being more highly expressed in elderly people and men, which would provide more opportunities for viruses to enter cells. As a result, the risk of serious clinical manifestation is higher in older adults than in the younger population [134]. CBD and THC reduce disease sensitivity by modulating ACE2 protein levels. Wang et al. [130] investigated the anti-inflammatory effects of cannabis extracts on tissues (Human 3D models: Epioral, EpiAirway, and EpiIntestinal) initially treated with pro-inflammatory cytokines. The extracts from *C. sativa* L., especially CBD, demonstrated downregulated *ACE2* gene expression.

A study of 93,565 patients in Chicago with SARS-CoV-2 found those who had previously taken CBD had a significantly lower incidence of SARS-CoV-2 infections compared to matched pairs or the general population [32]. Only 1.2% of patients prescribed with CBD contracted SARS-CoV-2 compared to 12.2% of matched, non-cannabinoid patients. Hence, CBD and its 7-OH-CBD metabolite appear to be potential preventive and treatment agents for SARS-CoV-2 in the initial stages of infection. The findings indicate that CBD blocked SARS-CoV-2 infection early in its course and reduced the risk of SARS-CoV-2 infection in humans. CBD and 7-OH-CBD strongly blocked replication of SARS-CoV-2 in the A549 human lung cancer cell line. CBD also blocked the expression of viral genes. Moreover, treatment also triggered interferon expression and activated the antiviral signaling pathway [32]. These results are in agreement with previous research works showing the ability of cannabinoids to inhibit the formation of cytokine in macrophages [135].

Additionally, the experiments have shown the positive action of CBD, which may help during COVID-19 infection [133]. Studies on mice have shown that CBD increases the level of apelin, which reduces inflammation and protects the lungs. It was found that CBD intake increased the blood apelin level by as much as 20 times and, importantly, this was accompanied by in an increase in oxygen level in the lungs.

Cannabinoids may possibly prevent SARS-CoV-2 infections. Cannabinoid acids isolated from *C. sativa* L. were revealed to be allosteric and orthosteric ligands with micromolar affinity for the SARS-CoV-2 spike protein. Cannabigerolic acid and cannabidiolic acid protected human epithelial cells from infection with pseudoviruses expressing the SARS-CoV-2 spike protein and prevented SARS-CoV-2 from entering the cells. Importantly, cannabigerolic acid and cannabidiolic acid were equally effective against the SARS-CoV-2 alpha variant B.1.1.7 and beta variant B.1.351 [136]. Classen et al. [131] showed that cannabigerol and cannabicyclol (20 µM), other cannabinoids of *C. sativa* L., blocked SARS-CoV-2 fusion in Vero cells. Both compounds inhibit SARS-CoV-2 spike protein-mediated membrane fusion, as shown by a virus-free reporter fusion inhibition assay and by monitoring syncytia formation in Vero reporter cells.

### 2.5. Current Cannabinoid-Based Drugs Approved by the FDA

Cannabinoids are used in medical applications. The modern pharmacological armamentarium now includes synthetic CBD and THC (dronabinol), nabiximols (THC:CBD = 1.08:1.00), and synthetic nabilone [137].

Cannabinoid-containing preparations are used to treat neurological diseases such as epilepsy (drug Epidiolex^®^/Epidyolex^®^), multiple sclerosis (Sativex^®^ i.e., nabiximols), pain (migraine), or tic disorders. Cannabinoids are also being trialed in neurodegenerative diseases such as Parkinson’s disease (PD), Huntington’s disease, amyotrophic lateral sclerosis (ALS), dementia, and dystonia [138].

#### 2.5.1. Epidiolex^®^ CBD Extract as a Drug for Epilepsy

For several years, cannabidiol (Epidiolex^®^/Epidyolex^®^) has been approved in the US and in Europe as an add-on therapy for the treatment of two forms of epilepsy that are often drug-resistant, Lennox–Gastaut (LGS) and Dravet syndrome [138]. The starting dosage of Epidiolex^®^ is 2.5 mg/kg by mouth twice daily (5 mg/kg/day). After one week, the dosage can be increased to a maintenance dosage of 5 mg/kg twice daily (10 mg/kg/day); the maximum recommended maintenance dosage is 10 mg/kg twice daily (20 mg/kg/day) [139].

Randomized, controlled, and open-label research works have been performed to assess the effect of Epidiolex (99% pure CBD oral extract) in patients suffering from resistant epilepsy. The findings have led to FDA approval and the inclusion of Epidiolex by the Drug Enforcement Agency in Schedule V of the Controlled Substances Act [140].

A retrospective review of 108 patients with refractory epilepsy taking Epidiolex was executed in a single center. Patients received Epidiolex as 100 mg/mL oral solution, from 2 to 10 mg/kg/day to tolerance or a maximum 25–50 mg/kg/day dose for up to 192 weeks, depending on the single center. In the final assessment, 75% of the patients continued to use Epidiolex. The most usual causes of termination were lack of effectiveness, raised seizure activity, deterioration of behavior, and sedation. At baseline, 47.2% of patients were also taking clobazam, and 39.2% of these patients were able to lower their clobazam dose. Additionally, 53% could stop or decrease the dose of at least one other antiseizure drug [141]. Another extensive research work, executed between 2014 and 2019 with the participation of 892 patients, found CBD to have a positive effect in patients with treatment-resistant epilepsies. All patients received 20 mg/kg oral cannabidiol daily or matched placebo for 14 weeks. Cannabidiol administration was connected with a sustained reduction in seizures for up to 192 weeks with an acceptable safety profile, and the achieved results confirmed that CBD can be employed in long-term treatment [142].

Some findings indicate that cannabidiol use has a risk of side effects [143,144]. A recent meta-analysis of nine studies found CBD use was connected with an elevated risk of several adverse events in epilepsy patients, like somnolence, diarrhea, decreased appetite, and elevation of alanine and aspartate aminotransferases [145].

#### 2.5.2. Sativex CBD Extract as a Drug for Treating Muscle Stiffness and Spasms Caused by MS

Sativex is the trademark of a cannabis drug called Nabiximols consisting of THC (2.7 mg per dose) and CBD (2.5 mg per dose). It has already been approved in Canada and most European countries for the treatment of moderate to severe spasticity in MS [57]. The license authorizes its use as a complement for treating MS. The dose requires titration until optimum symptom relief is achieved. This may take up to two weeks. The maximum licensed dose is 12 sprays per day [146].

Sativex users report dizziness and fatigue, however psychiatric symptoms, such as anxiety, mood changes, and paranoia, are rare [147]. Target symptoms were sleep disturbances, pain, and spasticity, with the most commonly reported side effects being drowsiness, emotional flattening, and difficulty concentrating [148].

However, at the doses generally used, Sativex did not affect driving ability and did not have any significant detrimental effects on cognition or mood; this was also noted in older people, as demonstrated by Rekand [149].

## 3. Limitations in *Cannabis sativa* L. Research and Use

Although the literature generally regards cannabis-based treatments as effective against neurological diseases [150,151,152], few studies have been conducted to assess the anti-inflammatory and anticancer properties of these compounds in humans: such research has been limited to in vitro and animal studies. While a few peer-reviewed clinical trials have been performed in humans, these have included only small numbers of patients [36,37]. 

It is important to note that translating preclinical research findings (from in vitro or animal studies) into clinical applications typically faces significant limitations.

Firstly, preclinical studies on cannabinoids are most commonly conducted on animals such as mice or rats, whose metabolism, immune systems, and physiology differ significantly from those of humans. It cannot be excluded that doses that are safe and effective in animals may be toxic or ineffective in humans. Additionally, the responses of the endocannabinoid system can vary between species, complicating the direct translation of results into clinical practice. 

One of the primary challenges faced by researchers is to determine the optimal therapeutic dose for humans. In preclinical studies, very high doses of cannabinoids are often used to achieve therapeutic effects. However, the clinical applicability of such doses is limited by their adverse effects, such as the psychoactive impact of THC. The lack of precise data on effective and safe dosing poses a significant barrier in the planning of clinical trials or applications. 

Another limitation is that many preclinical studies fail to account for the considerable variation in age, sex, lifestyle, diet, health status, genetics, and medications between patients. As such, it is difficult to predict the final effect of therapy. 

Furthermore, little data exist on the long-term effects of cannabinoid treatment. While most preclinical studies focus on the short-term therapeutic effects of cannabinoids, it is crucial to understand their long-term impact on the human body, including the potential side effects of treatment, as well as interactions with other drugs, and the risk of developing tolerance or dependency.

Although Epidiolex^®^ and Sativex^®^ are approved as drugs for neurological diseases, both have potential side effects such as mental depression or suicidal ideation, which should also be taken into consideration [138,153,154].

It is important to mention the other challenges associated with the therapeutic use of *L. sativa*, such as the need to standardize extracts or interactions with drugs and other cannabinoids. 

It is important to note that the composition of *C. sativa* extracts vary considerably in response to the variety and geographical location of the crop, as well as the method of isolation and standardization [14,15].

In addition, cannabinoids can inhibit efflux transporters and drug metabolizing enzymes and can possibly induce drug kinetic interactions with anticancer drugs. These interactions can enhance the cytostatic effect of the drugs and/or their side effects, or it may reduce the effective drug dose [155]. 

The interaction between cannabinoids themselves is also important. Indeed, a synergistic effect has been observed for the combination of CBD with cannabichromene (CBC) or THC. As a result, small concentrations can replicate much higher doses of the extract in inducing HNSCC cell death [156].

Also, the lack of appropriate legal regulations regarding the production and distribution of CBD and *C. sativa* L. oil products has led to considerable variation in the levels of CBD and tetrahydrocannabinol levels in the products [140]. 

Although the data presented in this article report the wide-ranging benefits of using *C. sativa* L. and cannabinoids, recreational use of marijuana continues to have negative consequences. Cannabis is the most commonly taken drug of natural origin, as evidenced by data from the report prepared by UNODC; about 269 million people used it in 2018 [157].

It is possible that the further relaxation of restrictions regarding the availability of medicinal marijuana may also result in an increase in health problems for users.

## 4. Conclusions

(1) *C. sativa* L. is an extraordinary plant that provides a valuable raw material for medical applications. Its secondary metabolites, cannabinoids, have attracted growing interest in the fight against illness, mainly due to their effect on CB1 and CB2 cannabinoid receptors.
(2) Cannabinoid-containing preparations are used to treat neurological diseases such as epilepsy, multiple sclerosis, and pain [150,151,152], and are also being trialed in neurodegenerative diseases e.g., in Parkinson’s disease or amyotrophic lateral sclerosis [138].
(3) Cannabinoids and other antioxidant compounds of *C. sativa* L., such as cannflavin A and B, exert antioxidant potential by neutralizing free radicals, supporting metal chelation, increasing GSH level, and influencing the activity of antioxidative enzymes (Table 1).
(4) They also possess anti-inflammatory properties, inhibiting the progression of inflammation associated with the NF-κB and Keap1-Nrf2 signaling pathways [78], and inducing T-regulatory cells [51,52,53,55].
(5) In addition, their antiapoptotic, antiproliferative, antiangiogenic, and antimetastatic properties make them attractive potential cancer medications (Table 2)
(6) Cannabinoids have demonstrated antiviral potential (Figure 4) by inter alia blocking of SARS-CoV-2 translation and replication by suppressing M^pro^ protease, and by downregulating the serine protease TMPRSS2 and inhibiting *ACE2* transcription [130,132].
(7) There is a need to build a higher number of clinical trials on humans. These studies should include larger groups of patients and should use greater standardization of products and research methodology. There is also a pressing need to understand the long-term effects and action of cannabinoids in various diseases and their interactions with other drugs.

## Figures and Tables

**Table 2:** Different mechanisms of anticancer activity of extracts and single compounds contained in *C. sativa* L.

Extract, Compounds /Concentrations	Tested System	Mechanism of Action	References
*C. sativa* L. extracts and cannabinoids induction of apoptosis			
CBD 3 μmol/L	A549, H460 human lung cancer cells	Upregulates *COX-2* and *PPAR-γ*	[97]
*C. sativa* L. extracts—30 μmol/L CBD—10 μmol/L	PC3 prostate cancer cells	Increase caspase 3/7 activity, upregulate *TP53* and *Bax* expression, and induce silencing of *RBBP6*.	[100]
CBD 3.125–50.0 μmol/L	HCC—Hepatocellular carcinoma cells	Enhances p53 activation via ER stress	[113]
Extract from *C. sativa* 50% and 100% of their corresponding IC_50_ values	Melanoma cell lines MM418-C1, MM329, MM96L	Increases pro-apoptotic markers (Bax) expression Decreases anti-apoptotic markers (*Bcl-2*-) expression	[103]
CBD and THC 6, 10, 15 μmol/L	A375, A2058 and SK-Mel-28 melanoma cell lines	Release of mitochondrial cytochrome c and activate caspase-3/7	[110]
Cannabinol (CBN)	A172 glioblastoma, HepG2 liver cancer and HCC1806 breast cancer cell lines	Downregulates p21 and p27 arrested cell cycle in G1 or S phase	[114]
CBD induction of autophagy			
CBD 2–20 μmol/L	Human cholangiocarcinoma cells (KKU-213B, KKU-100, KKU-055)	Upregulates LC3BII, downregulates p62, inhibits p-PI3K, p-AKT, and p-mTOR pathways	[101]
CBD 3, 6, or 10 μmol/L	Head and neck squamous cell carcinoma (FaDu, SNU899, SCC15, Hep2)	Increases in Beclin- and LC3II-coding gene expression	[115]
*C. sativa* L. extracts and cannabinoids induction of tumor regression			
*C. sativa* L. essential oil extracted from flowers and leaves	Lewis lung cancer grafted mice model	Inhibits tumor growth, decreases of TNF-α and IL-6, increases in CD4+, CD8+ T lymphocytes count	[116]
CBD 5 mg/kg BW	Mice C57BL/6 with B16F10 murine melanoma tumor	Reduces tumor size	[96]
CBD—10 mg/kg BW THC—10 mg/kg BW	NSG mice	Deplete tumor growth	[110]
Cannabigerrol (CBG) 2.5 mg/kg or 3.75 mg/kg	Tumor-bearing mice	Decreases tumor progression by combining CBG and αPD-L1, decreases colony-stimulating factor 1 (CSF-1) level	[102]
PHEC-66 extract from *C. sativa* L.	MM418-C1, MM329, and MM96L melanoma cell lines	Induces DNA fragmentation, and arrests cell progression at the G1 cell cycle checkpoint.	[103]
Cannabinoids decrease tumor proliferation.			
CBD and THC 3.9–500 µg/mL	A375 human melanoma cells	Inhibit ERK1/2 signaling pathway phosphorylation, which is responsible for the regulation of cell proliferation.	[104]
Cannabinol (CBN)	A172 glioblastoma, HepG2 liver cancer and HCC1806 breast cancer cell lines	Decreases cancer cell proliferation	[114]
Cannabinoids induce suppression of cell invasion/inhibition of angiogenesis			
R(+)-methanandamide analog 0.1 µmol/L THC—0.01 µmol/L	HeLa, C33A human cervical carcinoma, A549 human lung cancer cells	Increase expression of *TIMP-1*, which mediates an anti-invasive effect of cannabinoids.	[105]
Cannabinoid JWH-133 8 days at 50 µg/day	Mice with malignant gliomas	Inhibits expression of proangiogenic factors: vascular endothelial growth factor, and angiopoietin 2	[36]

## Abbreviations

AAE: ascorbic acid equivalents; ABTS: 2,2’-azinobis(3-ethylbenzothiaziline-6-sulfonate), BHT: butylated hydroxytoluene, CBC: cannabichromene, CBD: cannabidiol, CBDA: cannabidiolic acid, CBG: cannabigerol, CBGA: cannabigerolic acid, CBN: cannabinol, CFL-A: cannflavins A, CFL-B: cannflavins B, CFL-C: cannflavins C, COX-2: induced cyclooxygenase, CRP: C-reactive protein, ECS: endocannabinoids system, FBL-03G: cannflavin B isomer, FDA: Food and Drug Administration, FRAP: fluorescence recovery after photobleaching, HCC: hepatocellular carcinoma cells, LPS: lipopolysaccharide, MA: R(+)-methanandamide; MAPKs: mitogen-activated protein kinase, PPAR-γ: peroxisome proliferator-activated receptor gamma, THC: tetrahydrocannabinol, TIMP-1: inhibitor of metalloproteinase-1, TMZ: temozolomide, TNF-α: tumor necrosis factor α; TRE: treatment-resistant epilepsies.
